# Socioeconomic determinants and self-rated health among hotel housekeepers in the Balearic Islands (Spain)

**DOI:** 10.3389/fpubh.2024.1390582

**Published:** 2024-09-02

**Authors:** Xenia Chela-Alvarez, Alfonso Leiva, Oana Bulilete, Joan Llobera

**Affiliations:** ^1^Primary Care Research Unit of Mallorca, Balearic Islands Health Service, Palma, Spain; ^2^GrAPP-caIB – Health Research Institute of the Balearic Islands, Palma, Spain; ^3^RICAPPS- Red de Investigación Cooperativa de Atención Primaria y Promoción de la Salud - Carlos III Health Institute (ISCIII), Madrid, Spain

**Keywords:** self-rated health, hotel housekeepers, social determinants of health, lifestyles, occupational stress, financial strain, work–family conflict

## Abstract

**Background:**

Hotel housekeepers constitute an important occupational group in the Balearic Islands (Spain). Housekeeping is considered low-skilled and precarious and typically involves high physical demands and time pressure. The aim of this study is to analyze the association between the socioeconomic determinants of health and hotel housekeepers’ self-rated health.

**Methods:**

This is a cross-sectional study conducted in Primary Health Care in the Balearic Islands (November 2018–February 2019). Hotel housekeepers over 18 years of age with free access to the Balearic Public Health System who had been employed during 2018 were eligible.

**Results:**

We enrolled 1,043 hotel housekeepers; the mean score of health perceived status was 72.4/100 (SD 19.0). Those with a lower self-perceived health were statistically significant older, had Spanish nationality, lower level of studies, permanent or recurring seasonal contract, financial difficulties, a higher level of occupational stress, an external locus of control, reported work-life balance difficulties, were former smokers, insufficiently physical active and obese. We found lower scores in self-perceived health status score of −7.159 (CI95% -10.20- -4.12) among hotel housekeepers with osteoarthritis; −6.858 (CI95% -11.89- -1.82) among those with chronic depression; −3.697 (CI95% -6.08- -1.31) among those who reported difficulties in work-life balance; −2.414 (CI95% -4.69- -0.13) among participants who performed insufficient physical activity; −2.107 (CI% -4.44- -0.23) among those who reported financial strain. Lower self-rated health was also associated to a higher perceived stress, −1.440 (CI95% -2.09- -0.79); BMI (kg/m^2^), −0.299 (CI95% -0.53- -0.07); and longer time working as HH -0.177 (CI95% -0.33- -0.03).

**Conclusion:**

Our results underscore the importance of psychosocial (such as difficulties in work-life balance and occupational stress) and material factors (such as financial difficulties) when explaining differences in self-perceived health. Public health interventions aimed at improving health status must consider inequalities in material and working conditions.

## 1 Introduction

In the Balearic Islands, the tourist sector contributed to 44% gross domestic product (pre-pandemic data) (1), with hotel housekeepers (HHs) constituting an essential occupational group (around 13,000). Housekeeping is considered a low-skilled job, physically demanding, involving repetitive tasks, unhealthy postures, high demands, heavy workload and low control (2–9). These characteristics have been related to negative health outcomes, such as musculoskeletal disorders (MSD) (10, 11) and stress (2, 12, 13).

Health status is defined as a measure of how people perceive their health, including physical and mental health (14). Similarly, the World Health Organization defines the concept of health as a “state of complete physical, mental, and social well-being and not merely the absence of disease or infirmity” (15). Among the measures to assess health status, self-rated health (SRH) has been widely used and reported as a good indicator for health problems (16) and a strong predictor for mortality (17). Generally, individuals are requested to rate their health as excellent, very good, good, fair, or poor, or to evaluate it using a visual analog scale. Material, psychosocial and behavioral factors have been widely identified as key contributors to socioeconomic inequalities in health (18, 19). Consequently, individuals from lower socioeconomic status present higher rates of morbidity, mortality and lower SRH compared to their higher socioeconomic status counterparts (20, 21).

Understanding these disparities is crucial for addressing health inequalities. The framework of socioeconomic determinants of health (22, 23) emphasizes that the socioeconomic and political context in which people live influences their socioeconomic position.

The socioeconomic and political context includes the welfare state, governance, macroeconomic policy, social policies, public policy, cultural and societal values and epidemiological conditions. Although data from Southern Europe are relatively scarce (24), studies point to a high prevalence of poor SRH among the population, with no significant gender differences (25).

The second key component are structural determinants and socioeconomic position. Structural determinants play a decisive role in defining the person’s socioeconomic position (23). Research found that people in the lowest socioeconomic positions are more likely to report poor SRH (26–28).

Socioeconomic position affects health through intermediary determinants, which include material and psychosocial circumstances, as well as behavioral and biological factors (23).

As Solar and Irwin (23) note, “socioeconomic health differences occur when the quality of these intermediary factors are unevenly distributed between the different socioeconomic classes.” The main categories of intermediary determinants are: (i) *material/structural factors*, (i.e., housing conditions, financial problems, employment status and physical work conditions); (ii) *behavioral factors,* (i.e., smoking, alcohol consumption, dietary habits, physical activity and body mass index (BMI)); (iii) *psychosocial factors*, linked to life events, psychosocial stressors (e.g., job strain), social support and coping.

Income is considered a material factor within the framework of social determinants of health and has been strongly related to SRH. Financial strain has been described as a significant stressor, affecting both physical and emotional well-being (29, 30). Half of the workers in Southern Europe experienced financial strain and consequently are more likely to report poor SRH status (25, 31).

Other material factors include physical work conditions. In addition to the heavy workload, physical demands and repetitive tasks that involves hotel housekeeping, the total number of hours worked (both paid and unpaid) has been related to health outcomes (32). Women spending more than 36 h per week on housework are more likely to show lower SRH (33). Clearly, caregiving and housework entail exposure to ergonomic and psychosocial risks, primarily due to the physical and mental strain of caring for dependents (34). Kim and Cho (35) found that female workers with higher household burdens and caring responsibilities for older people had a higher prevalence of MSDs than those without such demands. Notably, the number of household members positively correlates with poor SRH in manual workers (36).

This highly demanding work, together with little control and few rewards (37–39), has been reported as a significant source of stress (2). In fact, most jobs in the hospitality sector have been associated with occupational stress (40). Further, higher levels of perceived occupational stress have been related to poorer SRH among workers with lower educational levels (41).

Social support is considered a psychosocial factor and is defined as ‘the degree to which a person’s basic social needs are gratified through interaction with others’ [(42), p. 147]. Lower social support has been associated with worse SRH (43, 44). Also, a study by Brønholt et al. (45) found that exposure to adverse psychosocial work factors (i.e., low social support, low skill discretion, job insecurity, etc.) increased with lower SEP and were associated with poor SRH in women and men.

Furthermore, work–family conflict (WFC), another psychosocial factor, occurs when the responsibilities of one life sphere interfere with the accomplishment of the responsibilities in other life domains (46, 47); that is, when paid work, caregiving and household demands become incompatible WFC is associated with poorer SRH (48–50), has been reported as higher among working-class people (51, 52) and is positively associated with health problems such as depression and poor physical health (24, 53).

The locus of control (LOC) refers to the perception a person has about the control of the events that occur in their life. It is regarded as a psychosocial variable because it encompasses both the psychological domain, reflecting individual beliefs, and the social domain, as it is influenced by social experiences (54). LOC can be internal (people who believe that what happens in their life is consequence of their attitudes, actions and behavior) or external (people who attribute what happens in their life to fortune, fate or others’ decisions) (54). Mounce et al. (55) found that an external LOC was positively associated with the development of multimorbidity and Kesavayuth et al. (56) found that people with an internal LOC had better SRH (both physical and mental).

Regarding behavioral factors, healthy lifestyles are associated with better SRH (57–59). However, a systematic review of Dieker et al. (26) concluded that “work factors seem to be important contributors to socioeconomic inequalities in SRH irrespective of lifestyle behaviors.” Similarly, Brønholt et al. (45) found that work factors explained almost half of the social inequalities in SRH.

Importantly, overweight and obesity have been associated with a reduction of years lived in good health (60) and with poorer overall health compared to having a normal weight (61). The systematic review carried out by Newton et al. (62) reported that “females of lower life course socio-economic status had significantly higher odds of obesity compared with those of higher life course socio-economic status”.

HHs’ health is of particular relevance due to their structural position within society as low skilled workers and as women. Thus, HHs should present a significant relationship between material and psychosocial factors and SRH. This population warrants attention because most existing studies focus on middle-class or highly educated and skilled occupational groups (24).

Building on the conceptual framework on the social determinants of health (23), this study aims to: (i) estimate the personal, occupational, psychosocial, and behavioral characteristics of HHs of the Balearic Islands, a low-status occupational group; and (ii) explore which factors influence their SRH.

## 2 Materials and methods

Cross-sectional study carried out in 39 out of 58 Primary Health Care Centres in the Balearic Islands between November 2018 and February 2019.

### 2.1 Subjects

HHs over 18 years of age who had health coverage in the Balearic Public Health System and worked as HHs during 2018 were included after signing the informed consent form. HHs with a language barrier were excluded.

An initial list of about 13,000 possible HHs was available from the Balearic Health Services and Balearic Public Employment Service; 978 HHs were needed to estimate population parameters, with a 3% precision and a confidence of 95%. A randomly sample of 1,043 HHs was selected; in case they did not accept or were not possible to contact them, they were replaced randomly from the initial list.

### 2.2 Data collection

Data collection was carried out through in-person interviews using a structured questionnaire. Each interview lasted approximately 1 h and was administered by trained nurses. The interviews were conducted in a consultation room at the Primary Health Care Center. Nurses were responsible for the recruitment of study participants: HHs were invited by telephone and if they met the inclusion criteria they were given an appointment in their closest Primary Health Care Center.

A total of 14 nurses were hired by the Project and involved in data collection. They underwent a 3-h training session to become acquainted with the project and learn how to conduct the interviews and administer the questionnaires. This training aimed at standardizing procedures, ensuring data consistency, and minimizing losses and errors.

In order to recruit the required number of HHs, 4,436 phone calls were made (some HHs were called more than once in order to be contacted or recruited). Finally, 1,043 HHs were enrolled: 773 in Mallorca, 89 in Menorca, 137 in Ibiza and 44 in Formentera.

### 2.3 Measures

#### 2.3.1 Dependent variable

Self-rated health: participants were asked to rate their overall health on the day of the interview on a 0–100 vertical visual analog scale (VAS), taken from the EuroQoL-5D-5L, a generic instrument for describing and valuing health (63, 64).

#### 2.3.2 Independent variables

##### 2.3.2.1 Socio-demographic and anthropometric

The sociodemographic characteristics were age, nationality and sex. The anthropometric variables were self-reported height and weight; after, BMI index (kilograms/m^2^) was calculated. Categories obtained were: below normal weight < 18.5; normal weight ≥ 18.5–24.9; overweight≥25–29.9; class I obesity ≥30–34.9; class II obesity ≥35–39.9; class III obesity ≥40.

##### 2.3.2.2 Material/structural factors

We asked about educational level, type of contract (permanent, recurring-seasonal or temporary), type of establishment (apartment, hotel, etc.), hotel category, number of rooms cleaned per day, years working as HHs, months worked last tourist season, and hours worked per week.

Financial strain was measured by asking *“A household might have different sources of income and more than a member can contribute with his/her income. Thinking about the total of your household monthly income, can you make ends meet…?”* with a 5 option Likert scale from “very easy” to “very difficult.” The question was taken from the EQLS 2016. Following previous studies, we dichotomized the answers (25); answers “with some difficulty,” “with difficulty” and “with great difficulty” were considered as suffering from financial strain.

##### 2.3.2.3 Behavioral factors

Smoking habits: the question had 4 options: do not smoke, former smoker (more than a year without smoking), former smoker (more than 6 months without smoking but less than a year), active smoker (1 or more cigarettes a day). This variable was transformed into a three categories variable (non-smoker, former smoker and active smoker).

Physical activity: we used the validated Spanish version of the Brief Physical Activity Assessment Tool (65), which identifies inactive patients (66). Two questions were asked about the frequency of performing 30 min of moderate and 20 min of vigorous physical activity a week. A total score of 4 or more was considered as sufficiently active (67). Their overall sensitivity in the detection of insufficient physical activity was 79.9% compared with accelerometer (68).

Healthy diet: we used a short, easy-to-administer, and validated screening questionnaire (69), with two questions regarding regular fruit and vegetable intake. A final score ranging from 0 to 10 was obtained: 5 or more points correspond to an intake of 5 servings of fruit or vegetables and thus considered as a healthy diet. The overall sensitivity in the detection of unhealthy diet was 76.8% (LR + = 3.1 and LR- = 0.31). Also, the correlation with the PREDIMED Food Frequency Questionnaire was R spearman 0.65 among women (69).

##### 2.3.2.4 Psychosocial factors

Locus of control (LOC) was assessed through the question *‘To what extent do you agree with the following sentence: «I feel what happens in my life is often determined by factors beyond my control?»*, with Likert-type response options ranging from 1 (completely disagree) to 6 (completely agree). A forward translation into Spanish of the measurement used by Mounce et al. (55) was made as recommended by Ortiz-Gutiérrez and Cruz-Avelar (70). Internality is considered when the respondent answers options 1 to 3 (disagreement statements).

Level of stress at work was measured with the question, *‘Overall, taking into account the conditions in which you do your work, indicate how you consider the level of stress at your work on a scale from 1 (very stressful) to 7 (not at all stressful)’*.

WFC was measured through the question ‘*How easy or difficult is it for you to combine work with your care responsibilities?*’, with four response options (from “Very easy” to “very difficult”), taken from the European Quality of Life Survey (EQLS, 2016) (51). We dichotomized answers (good work-life balance (WLB) and poor WLB), as done in other studies (71).

Social support was measured by DUKE-UNC-11 (72), an 11-item questionnaire to assess functional elements of social support (including confidant and affective support) validated in the Spanish population (73, 74). Each item is valued in a 5-points scale (ranging from 1 point: “much less than I would like,” to 5 points: “as much as I would like”). A final score ranging from 5 to 55 is obtained; 32 points or below correspond to low social support and more than 32 points to adequate social support (73).

Number of household members: HHs were asked if they lived on their own. In case of a negative response, participants were asked who they were living with. This variable was operationalized as number of people living in the household.

The distribution of domestic tasks and care of people were evaluated through the question ‘*At home, who is mainly in charge of household chores and the care of people who are not able of caring for themselves?*’. This question had to be answered in relation to four items extracted from the 2011–12 Spanish Health Survey: taking care of children under 15, taking care of a disabled person, taking care of people older than 74 years, and responsibility for domestic tasks (cleaning, ironing, cooking,…). For each item, the response options available were: HHs alone; HHs’ partner alone; shared with their partner; shared with a person other than their partner; another person living at home other than their partner; a hired person; a person who does not reside in the house; social services; him/herself; another situation; do not know/do not answer. It was a multiple answer question. In order to simplify the analysis and the results, for bivariate and multivariate analyses we transformed the care of dependents into a dichotomous variable: being in charge alone of a dependant or not (including the three items). The variable ‘main person in charge of domestic tasks’ was also dichotomized: being the main responsible vs. another situation.

##### 2.3.2.5 Self-reported morbidity

Participants were asked to indicate whether they had any of the following chronic diseases: arterial hypertension, diabetes, chronic depression, osteoarthritis, high cholesterol, chronic obstructive pulmonary disease (COPD), problems in thyroid, asthma, hemorrhoids, malignant tumor, osteoporosis, embolism, heart attack or cerebral hemorrhage, cirrhosis or liver dysfunction, and heart diseases (based on the Spanish National Health Survey).

### 2.4 Statistical analysis

Categorical variables are presented in absolute numbers, percentages and CI95%, while quantitative variables are presented as means and standard deviations (SD) or medians and interquartile ranges (IQR).

To assess the relationship between material, psychosocial, behavioral factors and chronic conditions with SRH the chi-squared test and Student’s t-test were used; *p-*values under 0.05 were considered statistically significant (2-sided tests). We only assessed the relationship with SRH for comorbidities with a prevalence greater than 2%.

Generalized linear model with a normal distributed dependant variable and identity link function was performed. Independent variables showing a statistical significance of *p* < 0.20 and potential confounding factors (smoking, BMI, fruit and vegetable intake, physical activity) were selected. Final predictive variables were selected from a backward stepwise procedure; Akaike’s information criterion AIC (75) and the Bayesian information criterion BIC (76) were used for model selection.

We further tested interactions of age and LOC with lifestyles and occupational stress.

SPSS for Windows version 23.0 and STATA version 13 were used for analyses.

## 3 Results

A total of 1,043 HHs were included. Their sociodemographic and material characteristics are displayed in Table 1. Most participants were Spanish, had completed compulsory education, worked under a recurring-seasonal contract and in a four-star hotel. The median of hours worked per week was 40 (IQR = 40.0–40.0) and the median of rooms cleaned per day was 18 (IQR = 15.0–22.0). More than a half indicated difficulties making ends meet.

**Table 1:** Sociodemographic, anthropometric and material characteristics of hotel housekeepers.

	MD	IQR
Age	43.0	36.0–51.0
	*n* (%)	CI 95%
Nationality (*n* = 1,043)		
Spanish	563 (54.0)	50.9–57.0
Double nationality	182 (17.4)	15.2–19.9
Foreign	268 (28.6)	25.8–31.4
Level of studies (*n* = 1,041)		
Illiterate/ primary incomplete	34 (3.3)	2.3–4.5
Compulsory education (primary and secondary)	591 (56.8)	53.7–59.8
Post-compulsory secondary education	298 (28.6)	25.9–31.5
University	118 (11.3)	9.5–13.4
Type of contract (*n* = 1,016)		
Permanent	63 (6.2)	4.8–7.9
Recurring seasonal contract	551 (54.2)	51.1–57.3
Temporary	402 (39.6)	36.5–42.6
Type of establishment (*n* = 1,043)		
Hotel	625 (59.9)	56.9–62.9
Apart-hotel	351 (33.7)	30.8–36.6
Rural hotel	42 (4.0)	2.9–5.4
Others	25 (2.4)	1.6–3.5
Hotel category		
1*	9 (1.0)	0.4–1.8
2*	37 (4.0)	2.8–5.4
3*	210 (22.5)	19.9–25.3
4*	574 (61.5)	58.3–64.7
5*	103 (11.0)	9.1–13.2
Making ends meet		
Easy	446 (43.3)	40.2–46.3
Difficult	585 (56.7)	53.7–59.8
	MD	IQR
Years working as hotel housekeeper	8.0	4.0–15.0
Months worked last season	7.0	6.0–8.0
Hours worked per week	40.0	40.0–40.0
Number of rooms/day cleaned	18.0	15.0–22.0

Psychosocial factors and lifestyles of HHs are shown in Tables 2, 3. The median of the level of occupational stress was 5.0 (IQR 4.0–6.0); most of the HHs had normal social support, an external LOC and WLB difficulties. Over 50% were non-smokers and insufficiently physically active. Nearly 80% of the participants ate less than 5 servings of fruit and vegetables a day, and almost 50% had normal weight.

**Table 2:** Characteristics regarding psychosocial factors of hotel housekeepers.

	MD	IQR
Level of occupational stress	5.0	4.0–6.0
	*n* (%)	CI 95%
Social support (*n* = 1,025)		
Low	65 (6.3)	4.9–8.0
Normal	960 (93.7)	92.0–95.1
Locus of control (*n* = 1,035)		
Internal	418 (40.4)	37.4–43.4
External	617 (59.6)	56.6–62.6
Number of household members (*n* = 1,043)		
1	57 (5.5)	4.2–7.0
2 or 3	576 (55.2)	52.1–58.3
4 or more	410 (39.3)	36.3–42.3
Person in charge of domestic tasks (*n* = 1,014)		
HHs alone	354 (34.9)	32.0–37.9
Sharing or another person in charge	660 (65.1)	62.1–68.0
Taking care for any dependant (≤15 y.o, ≥74y.o or disabled) (*n* = 551)		
HHs alone	136 (27.1)	23.3–31.3
Sharing or another person in charge	365 (72.9)	68.7–76.7
Work-life balance (*n* = 1,035)		
Easy	449 (43.4)	40.3–46.5
Difficult	586 (56.6)	53.5–59.7

**Table 3:** Characteristics regarding behavioral factors of HHs.

	*n* (%)	CI 95%
Smoking (*n* = 1,043)		
Non-smoker	595 (57.0)	54.0–60.1
Former smoker	127 (12.2)	10.3–14.3
Active smoker	321 (30.8)	28.0–33.7
Physical activity (*n* = 1,040)		
Sufficiently active	447 (43.0)	39.9–46.1
Insufficiently active	593 (57.0)	53.9–60.1
Fruit and vegetable intake (*n* = 1,041)		
<5 servings a day	819 (78.7)	76.1–81.1
≥5 servings a day	222 (21.3)	18.9–23.9
Body mass index		
Underweight	21 (2.1)	1.3–3.2
Normal	491 (49.0)	45.8–52.1
Overweight	329 (32.8)	29.9–35.8
Obesity class I	124 (12.4)	10.4–14.6
Obesity class II	28 (2.8)	1.9–4.0
Obesity class III	10 (1.0)	0.5–1.8

The mean of the SRH was 72.4 (SD = 19.0). Results of the association between sociodemographic, anthropometric, material, psychosocial and behavioral factors and SRH (Table 4) show that HHs with a lower SRH were older (*p* < 0.001), had Spanish nationality (*p* < 0.001), had attained a lower level of studies (*p* = 0.007), had permanent or recurring seasonal contract (*p* = 0.039), had been working as HHs for longer (*p* ≤ 0.001), had financial difficulties (*p* < 0.001), had a higher level of occupational stress (*p* < 0.001), an external LOC (*p* = 0.001), reported WLB difficulties (*p* < 0.001), were former smokers (*p* = 0.012), were insufficiently physical active (*p* = 0.040) and were obese (*p* = 0.002).

**Table 4:** Association between individual, material, psychosocial and behavioral factors with self-rated health.

	Self-perceived health status	
	$\bar{x}$ (SD)	*p*-value
Age		
<35 years	77.3 (1.1)	<0.001
35–49 years	72.04 (0.8)	
> 50 years	69.1 (1.1)	
Nationality		
Spanish	69.6 (0.8)	<0.001
Double	74.8 (1.3)	
Foreign	76.2 (1.1)	
Level of studies		
Compulsory incomplete	70.0 (3.8)	0.007
Primary and secondary compulsory	70.7 (0.8)	
Post-compulsory secondary	74.9 (1.0)	
Tertiary	74.7 (1.5)	
Type of contract		
Permanent	71.4 (2.9)	0.039
Recurring seasonal	71.1 (0.8)	
Temporary	74.2 (0.9)	
Type of establishment (*n* = 1,043)		
Hotel	72.8 (0.7)	0.473
Apartment	71.5 (1.1)	
Rural hotel	75.6 (3.1)	
Others	70.2 (3.7)	
Years worked as HHs		
0 to 4	76.0 (17.6)	≤0.001
5 to 9	73.6 (19.4)	
10 to 14	72.2 (18.7)	
15 to 19	66.5 (18.9)	
20 and over	67.2 (19.8)	
Average of months worked per year		
Up to 6 months	72.6 (18.7)	0.648
From 7 to 12 months	72.0 (19.4)	
Number of hours worked per week		
≤40 h	72.2 (19.4)	0.466
>40 h	73.2 (17.4)	
Number of rooms cleaned per day		
≤18 rooms/day	72.8 (19.2)	0.458
>18 rooms/day	71.9 (18.8)	
Making ends meet		
Easy	75.0 (18.5)	<0.001
Difficult	70.4 (19.1)	
Level of occupational stress		
Few stress (1 and 2)	80.3 (1.7)	<0.001
Medium stress (3 to 5)	73.9 (0.8)	
Very stressful (6 and 7)	68.7 (0.9)	
Locus of control		
Internal	76.3 (17.2)	<0.001
External	69.6 (19.7)	
Number of household members		
HHs alone	73.0 (2.3)	0.391
Two or three household members	71.6 (0.8)	
More than three household members	73.3 (0.9)	
Domestic tasks		
HHs is the main person in charge	71.4 (18.9)	0.328
HHs shares the responsibility	72.6 (19.0)	
Taking care for any dependant (≤15 y.o, ≥74y.o or disabled)		
She alone	18.9 (1.7)	0.220
Sharing or another person	18.2 (0.9)	
Work-life balance		
Easy	75.3 (19.1)	<0.001
Difficult	70.2 (18.6)	
Social support		
Normal	72.7 (18.9)	0.118
Low	68.7 (19.8)	
Smoking		
Non-smoker	73.7 (0.8)	0.012
Former smoker	68.5 (1.8)	
Active smoker	71.5 (1.0)	
Physical activity		
Insufficiently active	71.4 (19.7)	0.040
Sufficiently active	73.8 (17.7)	
Fruit and vegetable intake		
Non adherent	72.9 (18.4)	0.168
Adherent	70.8 (20.7)	
Body mass index		
Underweight	80.9 (3.2)	0.002
Normal	72.9 (0.8)	
Overweight	72.9 (1.0)	
Obesity	67.7 (1.7)	

Morbidity was significantly associated with SRH: those with arterial hypertension (*p* = 0.001), diabetes (*p* = 0.004), osteoarthritis (*p* ≤ 0.001) and chronic depression (*p* ≤ 0.001), COPD (*p* = 0.003), problems in thyroid (*p* = 0.007), hemorrhoids (*p* = 0.008) had lower levels of SRH (Table 5).

**Table 5:** Association between self-reported morbidity with self-rated health.

Chronic disease	Prevalence *n* (%)	Self-perceived health status		
		Yes $\bar{x}$ (SD)	No $\bar{x}$ (SD)	*p*-value
Arterial hypertension	109 (10.5)	66.10 (21.65)	73.11 (18.5)	0.001
Diabetes	27 (2.6)	60.22 (20.47)	72.70 (18.82)	0.004
Osteoarthritis	211 (20.2)	63.57 (19.46)	74.62 (18.18)	≤0.001
Chronic depression	60 (5.8)	61.12 (19.51)	73.06 (18.72)	≤0.001
High cholesterol	161 (15.4)	70.28 (18.13)	72.75 (19.10)	0.131
Chronic obstructive pulmonary disease	35 (3.4)	64.71 (14.03)	72.64 (19.06)	0.003
Problems in thyroid	63 (6.0)	65.32 (20.83)	72.83 (18.76)	0.007
Asthma	54 (5.2)	68.61 (18.39)	72.58 (18.98)	0.134
Hemorrhoids	70 (6.7)	65.94 (20.49)	72.83 (18.78)	0.008

### 3.1 Determinants of SRH

Lower SRH scores were observed among HHs: with osteoarthritis, mean difference: -7.159 (CI95% −10.20- -4.12); with chronic depression, −6.858 (CI95% -11.89- -1.82); who reported difficulties in WLB, −3.697 (CI95% -6.08- -1.31); who performed insufficient physical activity, −2.414 (CI95% -4.69- -0.13); who reported financial strain, −2.107 (CI% -4.44- -0.23). Lower SRH was also associated to a higher perceived stress, −1.440 (CI95% -2.09- -0.79); BMI (kg/m^2^), −0.299 (CI95% -0.53- -0.07); and longer time working as HH, −0.177 (CI95% -0.33- -0.03) (Table 6).

**Table 6:** Generalized linear model to analyze the association of sociodemographic, anthropometric, psychosocial, behavioral and material characteristics with self-perceived health status in hotel housekeepers, adjusted by age.

	Self-perceived health status		
	Mean differences	CI 95%	*p*-value
Osteoarthritis	–7.159	–10.20- –4.12	≤0.001
Chronic depression	−6.858	−11.89- –1.82	0.008
Difficulties in work-life balance	−3.697	−6.08- –1.31	0.002
Insufficient physical activity	−2.414	−4.69- –0.13	0.038
Financial difficulties	−2.107	−4.44- 0.23	0.077
Level of occupational stress (min 1- max 7)	−1.440	−2.09- –0.79	**≤**0.001
BMI	−0.299	−0.53- –0.07	0.010
Years worked as HHs	−0.177	−0.33- –0.03	0.021
≤5 servings of fruit and vegetables/day	1.994	−0.77- 4.76	0.158

Surprisingly, we found a negative association between the recommended fruit and vegetable daily intake and SRH status.

## 4 Discussion

This study analyses the relationship between SRH and sociodemographics, anthropometrics, material, psychosocial and behavioral factors as well as chronic diseases among HHs in the Balearic Islands. HHs mostly worked under recurring seasonal contracts (54.2%), with a median of 7 months worked during the 2018 season, underscoring the financial insecurity of this occupation. Regarding domestic tasks, more than one third reported to be the main person in charge, and almost one third indicated to take care herself alone for dependants. More than half HHs reported difficulties in WLB and making ends meet. One third were active smokers, almost two thirds were insufficiently physically active and more than 75% ate less than five servings of fruit and vegetables a day.

After controlling for confounding factors, we found a reduction in SRH status score among HHs with osteoarthritis, chronic depression, with difficulties in balancing work and life spheres, performing insufficient physical activity, reporting financial strain, and as levels of stress, BMI and years worked as HHs increased.

Besides this, the relevance of this study lies on focusing on a lower-status occupational group, since most previous studies report on skilled professionals (77).

The mean score of SRH is lower compared to that among the Balearic Islands general population: 72.4 (SD 19.0) vs. 75.99 (SD 19.71) (78). Moreover, the mean score among HHs is lower in all age groups compared to the reference norms in Spanish women reported in Hernandez et al. (79). These differences are higher in the youngest groups: HHs younger than 35 years old reported a mean score of 77.3 (SD 1.1) while the score was 84.5 to 87.0 among women between 18 and 34 years old. The high physical demands (3) and high work pace (2, 13) could partly explain this difference. Notably, the systematic review carried out by Dieker et al. (26) revealed that workers with a low socioeconomic status were at higher risk of poor SRH.

Our results indicated that chronic disease was associated with a worse SRH, in line with several studies. Higher number of chronic conditions have been associated with worse SRH (79) and quality of life (80) Concretely, Van Schoor et al. (81) found that clinical osteoarthritis was associated with fair-to-poor SRH, partly mediated by physical function. Val Jiménez et al. (82) identified a weak but negative correlation between the number of articulations affected by osteoarthritis and the SRH status ratings in the EuroQoL VAS.

Also our results indicated a negative relationship between SRH and depression. Previous studies found that, after adjusting for confounding variables, people rating their health as poor to fair were at higher risk of depression and anxiety compared to those who rated their health as good to excellent (83, 84). Notably, low socio-economic status and job strain has also been found to be associated with higher prevalence of depression (85–87).

Chela-Alvarez et al. (88) found that HHs identified stress, anxiety and MSD as the main occupational health problems. Results of Hsieh et al. (13) revealed that Latina HHs working in the United States usually experienced stress. Similarly, in the qualitative study of Chela-Alvarez et al. (2) conducted in the Balearic Islands, HHs emphasized the occupational stress derived from the high demands, and the lack of resources and little control (mainly because of incompatible demands) over their job. Also, this stress increased their levels of anxiety. Several studies carried out among workers found a negative relationship between experiencing stress and SRH (41, 89). Further, Nappo (90) found a negative relationship between working speed and SRH.

Similar to our results, several studies found a statistically significant association between WFC and poorer SRH (24, 48, 1). Despite this, some others suggest that poor SRH precede WFC (49). Lunau et al. (50) found that odd ratios adjusted (aORs) for sociodemographic, occupational, working conditions and household characteristics showed that women with poor WLB were twice more likely to report poor SRH.

Moreover, Nappo (90) found that workers whose job prevented them from devoting the time they wanted to their family and those who keep worrying about work during non-working hours (two components of WFC) were more likely to have low SRH. Studies identified a stronger relationship between WFC and suboptimal SRH also in highly educated women (48); however, results of our study among a low skilled and less educated group corroborate that WFC is an important factor that negatively affects SRH. Although Mensah and Adjei (71) found a higher prevalence of poor WLB in men compared to women, the aOR measuring the association between poor WLB and poor SRH among workers were slightly higher for women [aOR =2.21; CI95%: (1.99–2.45) and for men aOR = 1.97; CI95%: (1.78–2.18)]. Longitudinal studies are still needed to establish the direction of causation.

The proportion of HHs reporting insufficient physical activity outside work (57%) was higher than that among Spanish unskilled workers (41.5%) (91). We hypothesize that the physical tiredness of the HHs’ job prevents them from engaging in physical activity outside work or compensates low levels of physical activity outside work. Moreover, while physical activity has been associated with health benefits (92), worldwide the prevalence of insufficient physical activity is higher among women (93). Consistent with our results, Jepsen et al. (94) found that light or strenuous physical activity decreased the likelihood of fair/poor health; and van Oostrom et al. (41) found that workers with a low educational level who performed insufficient physical activity and were overweight or obese were more likely to report poor health. Also, Zarini et al. (59), after adjusting for age, lifestyles behaviors, ethnicity, health insurance and comorbidities, found that women who were physically inactive were more likely to report poor/fair SRH compared to those physically active. Additionally, Van der Berge et al. (95) found that workers in demanding physical activity occupations, obese and performing low levels of leisure time vigorous physical activity report lower levels of SRH. This association has been explained by “the physical activity paradox” (96): the negative effects of occupational physical activity on workers’ global health.

Data from the Spanish National Health Survey (91) indicated that the prevalence of obesity among unskilled workers (24%) was eight points higher than that described among HHs of this study. The daily physical demands of hotel housekeeping might partly explain this lower proportion of participants with obesity. In our study, after adjusting for age, chronic health conditions, and behavioral, psychosocial and material characteristics, BMI was associated negatively with SRH. Similarly, Hellgren et al. (97) reported a negative association between BMI and SRH at different ages and in both sexes, and independent of comorbidities. Also, Busutil et al. (98) found that, after controlling by chronic diseases, people with obesity reduced EuroQoL’s VAS score by an average of 1.9 and 3.7 points, compared to those with normal weight. And results of the longitudinal case–control study of Hulman et al. (99) indicated that poor SRH was associated with a higher BMI.

Our findings also indicated that financial strain affects negatively HHs’ health status, accordingly to previous research (100–21). Other studies have found a relation between indebtedness and a lower probability of reporting good or very good health (51, 102). Additionally, the study Artazcoz et al. (25) revealed that, after adjusting for age, migrant status, occupational and household characteristics, almost half of the workers in Southern European countries suffered financial strain, which was related to poor SRH and to low psychological well-being. Also, Hsieh et al. (13) revealed that sources of stress among Latina HHs working in the United States were work and family finances.

In our study, the proportion of active smokers (30.8%) was 10 points higher than daily and occasional smokers among Spanish unskilled workers (91). The inverse association between socioeconomic status and smoking has been described, but the work of Martinez et al. (103) included four latent variables (social cohesion, financial strain, sleep disturbance and psychological distress) in the association and found that higher socioeconomic status was negatively related to financial strain, and this “led to an increase in both sleep disturbance and psychological distress, which both resulted in an increase of smoking.” Also, higher socioeconomic status was related to an increased social cohesion, which made decrease smoking habits. The high proportion of HHs who reported financial difficulties and the stress related to their job might be an explanation for this higher proportion of smokers in our study.

Only 21.3% of our sample reported having five or more servings a day of fruit and vegetables; like in the other healthy behaviors, people with higher education levels are more likely to consume more fruits and vegetables (104). There are few studies to analyze the association between fruit and vegetable intake and SRH. In the living from Health program study, they found a positive relationship between fruit and vegetables intake and SRH (59). Our results indicated an inverse association between eating five or more serving of fruit and vegetable and SRH. In this cross-sectional study reverse causation might explain this counterintuitive relationship: people with lower scores of SRH being more aware of a healthy diet.

Despite performing insufficient physical activity and higher BMI, we did not found that lifestyle factors were related to a better SRH. The systematic review by Dieker et al. (26) concluded that socioeconomic inequalities in SRH depended on work factors, irrespective of the lifestyle.

Our findings suggest the importance of adapting interventions to the socioeconomic characteristics of the targeted population and focusing on the improvement of material and psychosocial factors more than behavioral in order to improve health status.

Public health decision makers must take into consideration all these variables when designing interventions to improve HHs’ health. Our results underscore the importance of health conditions, material and psychosocial factors; thus, interventions aimed to improve health status must be adapted to different socioeconomic groups. Also, governments should audit and regulate stress levels in companies. Further, policies and programs aiming to decrease financial strain among lower socioeconomic status families should be strengthen; these might include facilitating access to housing (social housing, subsidies…), enhancing public transport, as well as widening the offer of affordable physical activities. Moreover, since HHs work more intensely during summer months, availability of subsidized summer schools for their children should reduce WFC.

## 5 Strengths and limitations

As far as we know, this is the largest study conducted among HHs. The sample of 1,043 HHs is sufficient to guarantee its sensitivity and the randomization guarantee its representativeness of the population of HHs in the Balearic Islands. However, there is a limitation inherent to cross-sectional studies: results are susceptible to reverse causality, as we already mentioned in the discussion section, and causation cannot be proved. Also, data were collected in winter, when most HHs do not work or have less workload, since they were more available to participate; this situation might have influenced the results regarding SRH. Furthermore, data collection took place before the COVID19 pandemic, which has probably altered the situation to some degree.

Regarding the dependent variable of our study, the advantages of the EuroQol VAS in determining SRH include its simplicity, ease of administration, and straightforward scoring. However, the EQ-VAS relies on subjective evaluation based on an individual’s self-assessment of their health status, which can be influenced by factors such as locus of control, age, and educational attainment (105). Additionally, some studies have found that the EuroQol VAS is less sensitive and less responsive to changes in clinical outcomes than the completed EuroQol-5D (106). Lastly, work stress was assessed using a single question. This approach may not capture the full complexity of work stress experienced by individuals in specific occupations. Using a more comprehensive work stress scale would have provided a more accurate measurement. Therefore, the reliance on a single-question measure should be acknowledged as a limitation, as it may not fully reflect the multifaceted nature of occupational stress.

Finally, data have been collected in just one region of Spain. Housekeeping is a precarious occupation because of the temporality of the economic activity and because it is considered an unskilled job. However, in the Balearic Islands the trade unions have achieved better occupational conditions than other regions. For instance, in the Balearic Islands outsourcing is not allowed and hotel housekeepers work with a contract and not per hours, characteristics that have to be taken into account when comparing our results with those obtained in other settings.

## Data Availability

The datasets presented in this study can be found in online repositories. The names of the repository/repositories and accession number(s) can be found at: https://doi.org/10.5281/zenodo.10610378.
